# “We start where we are”: a qualitative study of barriers and pragmatic solutions to the assessment and reporting of implementation strategy fidelity

**DOI:** 10.1186/s43058-022-00365-4

**Published:** 2022-10-29

**Authors:** Christopher F. Akiba, Byron J. Powell, Brian W. Pence, Kate Muessig, Carol E. Golin, Vivian Go

**Affiliations:** 1 RTI International, Research Triangle Park, NC USA; 2 Center for Mental Health Services Research, Brown School, Washington University in St. Louis, St. Louis, MO USA; 3 Division of Infectious Diseases, John T. Milliken Department of Medicine, School of Medicine, Washington University in St. Louis, St. Louis, MO USA; 4 Center for Dissemination & Implementation, Institute for Public Health, Washington University in St. Louis, St. Louis, MO USA; 5 Department of Epidemiology, Gillings School of Global Public Health, University of North Carolina at Chapel Hill, Chapel Hill, NC USA; 6 Department of Health Behavior, Gillings School of Global Public Health, University of North Carolina at Chapel Hill, Chapel Hill, NC USA

**Keywords:** Implementation strategy fidelity, Specification, Tracking, Mechanisms

## Abstract

**Background:**

Fidelity measurement of implementation strategies is underdeveloped and underreported, and the level of reporting is decreasing over time. Failing to properly measure the factors that affect the delivery of an implementation strategy may obscure the link between a strategy and its outcomes. Barriers to assessing and reporting implementation strategy fidelity among researchers are not well understood. The aims of this qualitative study were to identify barriers to fidelity measurement and pragmatic pathways towards improvement.

**Methods:**

We conducted in-depth interviews among researchers conducting implementation trials. We utilized a theory-informed interview approach to elicit the barriers and possible solutions to implementation strategy fidelity assessment and reporting. Reflexive-thematic analysis guided coding and memo-writing to determine key themes regarding barriers and solutions.

**Results:**

Twenty-two implementation researchers were interviewed. Participants agreed that implementation strategy fidelity was an essential element of implementation trials and that its assessment and reporting should improve. Key thematic barriers focused on (1) a current lack of validated fidelity tools with the need to assess fidelity in the short term, (2) the complex nature of some implementation strategies, (3) conceptual complications when assessing fidelity within mechanisms-focused implementation research, and (4) structural issues related to funding and publishing. Researchers also suggested pragmatic solutions to overcome each barrier. Respondents reported using specification and tracking data in the short term until validated tools become available. Participants suggested that researchers with strategy-specific content expertise lead the way in identifying core components and setting fidelity requirements for them. Addressing the third barrier, participants provided examples of what pragmatic prospective and retrospective fidelity assessments might look like along a mechanistic pathway. Finally, researchers described approaches to minimize costs of data collection, as well as more structural accountability like adopting and enforcing reporting guidelines or changing the structure of funding opportunities.

**Discussion:**

We propose short- and long-term priorities for improving the assessment and reporting of implementation strategy fidelity and the quality of implementation research.

**Conclusions:**

A better understanding of the barriers to implementation strategy fidelity assessment may pave the way towards pragmatic solutions.

Contributions to the literature

- Implementation strategy fidelity, while essential in interpreting implementation research findings, is not often reported in implementation research
- Researchers conducting implementation trials reported barriers to assessing and reporting implementation strategy fidelity and identified opportunities for improvement
- This study identifies potential paths forward to addressing barriers to implementation strategy fidelity assessment, which may facilitate higher-quality implementation research

## Background

Implementation strategy fidelity is the extent to which a strategy is carried out as it was designed [1]. Given that many implementation strategies focus on behavior change, methods to determine their fidelity often mirror fidelity assessment of interventions (e.g., assessing the frequency, duration, or coverage of a strategy’s content) [1]. Implementation strategy fidelity differs from intervention fidelity regarding the level at which fidelity is assessed. While intervention fidelity may focus on assessing a social worker’s adherence to a psychosocial intervention, implementation strategy fidelity might focus on a facilitator’s adherence to key facilitation techniques meant to improve aspects of that social worker’s counseling. Like intervention fidelity, implementation strategy fidelity plays a crucial role when interpreting implementation trial results. First, fidelity assessment facilitates the evaluation of a Type III research error: failure to implement an intervention or strategy as intended, leading researchers towards an erroneous conclusion that null results are due to intervention or strategy characteristics, rather than to mal-implementation [2]. Second, in intervention research, fidelity moderates the relationship between an intervention and its main outcomes such that efforts carried out with greater fidelity tend to yield more desirable clinical outcomes compared to those carried out with lower fidelity [3, 4]. The same relationship may occur between implementation strategies and their outcomes of interest. While the assessment of a Type III error and fidelity moderation analyses are important for interpreting research findings, implementation strategy assessment is not well developed nor documented through routine reporting [1, 5]. Lack of consistent implementation strategy fidelity assessment and reporting challenges the field’s ability to compare strategies across studies or to replicate them [6–9]. Reviews note that an increase in trials that measure implementation strategy fidelity could improve understanding of how, why, and under what circumstances an implementation strategy impacted an outcome [8, 9]. Despite this potential benefit, barriers are not well understood [10]. In this qualitative study, we interviewed implementation researchers to gain an understanding of these barriers and potential opportunities for improvement.

## Methods

This qualitative study utilized a multi-stage purposive sampling strategy, combined with the theoretical domains framework (TDF), and reflexive-thematic analysis to elicit, categorize, and connect key barriers and pragmatic solutions to implementation strategy fidelity assessment and reporting.

We aimed to enroll implementation researchers who could describe barriers and solutions to assessing and reporting implementation strategy fidelity and the ways that those barriers and solutions affected decisions regarding whether or how to do so in current or recently completed implementation trials. Researchers were given a $50 gift card for participating. We designed a sampling approach that prioritized implementation research experience and diversity regarding health outcomes studied, study site geographic location, and research institutions. We combined three methods to create a sample frame of information-rich participants [11]: a search of principal investigators funded for active implementation trials using online funding databases (e.g., NIH RePORTER, CIHR Knowledge Translation and Commercializing); a literature search for first, second, and senior authors on manuscripts and protocols of recent implementation trials (search criteria included keywords like “implementation strategy, trial, evidence-based intervention”); and an assessment of leadership among implementation organizations (e.g., National Implementation Research Network, the Society for Implementation Research Collaboration). Eligibility criteria included any researcher identified through these 3 means. We then rank-ordered researchers based on the frequency with which they appeared across the 3 search strategies and approached them in order via email. Once interviews began, we utilized snowball sampling after each interview, asking participants if they would suggest other investigators we should consider including. We then re-ranked the researchers in our sample frame. Although we did not include an assessment of implementation strategy fidelity as one of our eligibility criteria, each participant described conducting some implementation strategy fidelity assessment in their own work. We set a target sample size of 20 researchers anticipating that the sample size would yield saturation [12].

Widely applied in research focused on identifying barriers and solutions, the TDF supplies researchers with 14 domains that encompass cognitive, affective, social, and environmental influences on behavior [13–20]. To best utilize the TDF, we first performed a literature search to understand known barriers to intervention fidelity assessment and reporting as well as their solutions. Examples included a lack of fidelity assessment knowledge, a lack of environmental influences like fidelity-focused publication requirements, and insufficient material resources required for fidelity assessment [4, 21, 22]. Solutions included a universally agreed-upon definition of fidelity, empirical approaches to fidelity assessment, and fidelity assessment requirements from funders and publishers [4, 21]. We categorized each barrier and solution identified into a TDF domain to create a semi-structured interview guide focused on exploring barriers and pragmatic solutions regarding implementation strategy fidelity assessment and reporting. After pilot testing the interview guide with a co-author (BWP), interviews were conducted by the lead author (CA) via video conference and transcribed verbatim by combining voice-to-text software and traditional transcription. Transcripts were not returned to participants for comment due to the high quality of transcription.

Data were analyzed using reflexive-thematic analysis [23]. This analytic approach includes data familiarization, codebook creation, coding, and theme generation [23, 24]. The TDF was further utilized to facilitate the creation of an initial codebook. While we developed some codes a priori, we created additional codes throughout the analysis process based on participants’ responses. For example, we describe in the section below how midway through our interviews we began asking participants what information they might need to feel assured that a strategy was delivered as intended when acting as a reviewer of implementation manuscripts. To categorize these responses, we developed codes like “implementation strategy fidelity assessment best practices” and “implementation strategy fidelity reporting best practices.” To better understand the connections between coded data, we augmented our coding process with memo-writing [25]. Utilizing the technique of “code weaving,” we connected salient words and phrases from our codes into our memo-writing. The review of coded material and their fit within memo categories combined to develop key themes [25].

All interviews were coded by the lead author (CA, a male graduate student), who met with co-authors throughout the coding and memo-writing process to ensure consistent code application and develop, define, and refine themes. No participants were interviewed more than once and they did not provide feedback on findings. Coding was carried out using Dedoose software v4.12.

This study was approved by the University of North Carolina Office of Human Research Ethics (IRB# 20-3718) and funded by the University of North Carolina at Chapel Hill Center for AIDS Research (P30 AI50410). Publication support also came from the Fogarty International Center (5D43TW011548-02), the National Institute on Drug Abuse (R01DA047876), the National Institute of Mental Health (5U19MH113202-05).

## Results

Interviews were conducted between June, 2021, and January, 2022. Our final sample included 22 researchers (66% of those invited), and interviews lasted 50 min on average. The sample comprised 18 faculty members from research-intensive universities, 2 from non-profit research organizations, 1 from a pharmaceutical company, and 1 US government implementation researcher. Nine researchers mostly centered on mental health and substance abuse outcomes, 8 on the delivery of general health services, 2 on HIV and ART care, 1 on cancer outcomes, 1 on non-communicable disease outcomes, and 1 on nutrition outcomes. Twenty-one participants were based in the US and the other participant in the UK. Eighteen focused their research domestically and 4 focused on low- and middle-income countries.

Our analysis identified four major themes: (1) a current lack of validated fidelity tools with the need to assess fidelity in the short term; (2) complexity of implementation strategies creating inherent difficulties assessing their fidelity; (3) conceptual complications when assessing fidelity within mechanisms-focused implementation research; and (4) structural barriers related to funding agencies and publication. We present each thematic barrier alongside proposed solutions using illustrative quotes to highlight key facets and variations within each theme. Solutions to barriers included (1) utilizing strategy specification and tracking techniques as well as theories of change, (2) allowing experts to lead the way in the development of fidelity tools of complex strategies, (3) adopting and enforcing implementation strategy fidelity reporting guidelines, (4) focusing funds for developing approaches to implementation strategy fidelity measurement, (5) utilizing technological innovations to facilitate efficient implementation strategy fidelity data collection, and (6) integrating implementation strategy fidelity assessment into mechanisms-focused implementation research.

### Barrier 1: Operationalizing implementation strategy fidelity

The vast majority of participants defined fidelity of implementation strategies as the extent to which a strategy was delivered as intended. When asked more specifically about how fidelity of implementation strategies ought to be assessed, participants provided a range of responses. Some described a desire for validated measures of implementation strategy fidelity akin to other implementation outcomes:

You know, I’ve seen some of the more recent literature around where they’ve now had validated measures for feasibility and acceptability, it would be nice if there was a more validated universal measure [of implementation strategy fidelity]…I think this is particularly challenging because it’s very individual to your own strategy which can be very significant.

Others described a preference for fidelity assessment using study-specific process measures but grappled with thoughts regarding their rigor.

I think the perception is that this is like tracking data, especially the process stuff, people don’t see it as a hard outcome. Unless it’s framed as fidelity ahead of time, and there’s so much in the process of tracking, there’s so much detail, there’s not one score of fidelity right? It isn’t a measure that’s easy to stick into a manuscript as another outcome.

The two participants quoted above described differing views regarding how researchers in our sample approached the assessment of implementation strategy fidelity. The first describes a desire for more rigorous, validated, universal tools that assess implementation strategy fidelity as an outcome variable. The second participant mentions the utility of tracking and process data to describe how a strategy was implemented. However, they question whether other researchers see process and tracking data as a “hard outcome,” suggesting others may perceive those data as less rigorous, and possibly of less scientific value. Several participants ultimately described how the development of validated strategy-specific fidelity tools may serve as a long-term goal but described the immediate utilization of process data as a pragmatic means of assessing implementation strategy fidelity in the short term. The variation regarding conceptual approaches to implementation strategy fidelity assessment may reflect the current state of implementation research. Another researcher expanded on this concept by describing how they approached implementation strategy fidelity with flexibility when serving as a peer reviewer:

Even if I’m not calling it implementation strategy fidelity it’s hard for me to imagine that someone would get to the publication phase and be like, ‘oh no, I don’t know, did I deliver the strategy?’ You know? I feel like there are ways that people could retrospectively piece together some kind of quality assurance metric…I mean, because I know that there aren’t established tools, I’m going to be a little bit less stringent [as a reviewer] about like ‘oh you’re not using a gold standard instrument’ if it doesn’t exist.

The participant quoted above was not alone in their approach to peer review of implementation research. As our interviews went on, we asked participants what would convince them, as reviewers, that strategies were delivered as intended. The majority of researchers shared the approach described by the participant above, with some additionally noting the utility of time-and-motion and costing data to describe the extent to which a strategy was implemented as designed. In the section below, we describe the time-intensive labor involved in developing rigorous, strategy-specific, fidelity tools. Given the immediate and ever-present need to assess the likelihood of a Type III error in implementation research, participants highlighted the value of process data to describe implementation strategy fidelity, despite some participants’ perceptions that it may have less rigor compared to the ideal of a validated fidelity tool. However, participants’ expectations that other researchers use process data to describe implementation strategy fidelity in their manuscripts signal its importance, even if “there’s not one score of fidelity.”

### Barrier 2: Implementation strategy complexity

Nearly all respondents remarked that as strategies become more complex, so too do their fidelity assessments, serving as a major barrier to routine measurement. When asked to describe what they mean by ‘complex strategies,’ almost all participants mentioned that complex strategies include a high volume of discrete strategies, and strategies that hinge on a more subjective interpersonal relationship between actors and action targets. Proposed solutions included the need for researchers with strategy-specific expertise to guide the field in fidelity assessment over the long term, and again, the utilization of process-like specification and tracking data to assess fidelity in the short term. When asked to describe specific complex strategies, participants frequently mentioned strategies like coaching, champions, and facilitation as the most complex implementation strategies. Several researchers described the additional frustration, and the feeling of being overwhelmed, when they think about assessing fidelity of complex multifaceted strategies:

I’ve read some articles and people are like ‘we specified an implementation strategy’ and they select like 2325 ERIC strategies [26]! And it’s like, you’re going to say we have to measure fidelity to each one? …I think people are just a little bit overwhelmed at unpacking the black box.

In addition to the encumbrance of assessing fidelity to multifaceted strategies, most participants also described the subjective nature of some strategies that hinge on interpersonal interactions, further complicating their fidelity assessment. One participant noted:

How much interaction is there between the strategy and the actor? How much discretion does the actor have over the execution of the strategy? And I think the more discretion that actor has, as with say facilitation or championing, some of those strike me as more art than science. So, when you have more art, how do you measure art? But when you have something where there isn’t as much discretion and it’s just ‘do this thing’ then it’s easy to measure that thing.

When participants were asked how they might approach assessing interpersonal aspects of implementation strategies, responses varied with respect to both methodologic approach and intensity. Several suggested adapting existing measures:

…One of the more widely used is a working alliance inventory, 12 items, right? Three subscales. ‘Do we agree on goals for what we’re doing?’ ‘Do we agree on the steps we take?’ and ‘Do we like each other,’ right?…Those could be translated pretty easily to [assess fidelity of] implementation strategies as well.

Others described a preference for assessing interpersonal facets of implementation strategies through qualitative interviews:

I definitely have a little bit more of a bias towards qualitative interviews for things like that, because I think that there’s a quality of the way that people talk about that relationship that you can kind of hear, you know? …It’s the type of relationship that they had with the facilitator…Like what are the things that organically come up for that participant as being meaningful to them that I think are harder to capture in a pre-specified survey.

Another researcher described their preference for assessing facilitation strategy fidelity by coding facilitators’ notes:

You know, do you have your facilitators fill out field notes or lab manuals? Or do they write down reflections of what they did every day with a site or with a group of people or every week? And could you code those to describe exactly what was done?

Several respondents also described their approaches to assessing facilitation fidelity, with one participant describing a method of recording facilitation sessions and scoring facilitators based on 4 components using a binary response option. Another described utilizing mixed methods, combining the use of time tracking logs and qualitative interviews to assess facilitators’ adherence to 20 core components. The differing approaches regarding quantitative and qualitative methods, the number of identified facilitation components, and varied response options echoes our first theme focused on how a research environment that lacks consensus on fidelity operationalization gives rise to varied approaches to fidelity assessment of the same implementation strategy.

When asked to describe the way forward for assessing fidelity of complex implementation strategies, responses fell broadly into two sub-themes. One set of responses focused on an approach utilizing the knowledge of experts who study specific complex strategies to guide the field forward by (1) identifying core components of various complex strategies, or even components of the same complex strategy given their broad nature, and (2) forming fidelity criteria to the identified components. The second focused on the importance of adequately specifying and tracking the distinct components of complex strategies and linking strategy activities to a theory of change.

Several participants suggested allowing experts to guide the way to fidelity assessment of complex strategies. These researchers felt that those most focused on any one complex strategy might be most knowledgeable regarding identification of strategy core components and how to assess fidelity to them.

I think it’s probably up to the people who are trying to develop the evidence, based on those strategies to try to figure this stuff out and I don’t think it’s lost on them, and I think that folks are doing it…The folks who are developing these strategies, it likely should be their job to think about [fidelity assessment of those strategies].

Two participants in our sample described their approach to developing a facilitation fidelity tool based on a scoping review and convening of experts to reach consensus of core components, followed by primary data collection to ascertain optimal fidelity data collection modalities for each component.

In the absence of developed fidelity tools, participants again described the utility of clarifying exactly how a strategy should operate (specification) and reporting on how it unfolded (tracking) to adequately determine if a complex strategy was implemented as intended. Researchers additionally described the importance of behavior change, organization, or implementation theories and frameworks in specifying the relationship between core activities within complex strategies and linking them to specific outcomes. Participants discussed how a theoretical rationale could give way to clarified strategy components and mechanistic pathways, and therefore clarified fidelity assessment. Respondents felt that utilizing a theory of change and specifying and tracking complex strategies might provide researchers with the tools to adequately determine if a strategy unfolded as it was designed.

### Barrier 3: Mechanisms and implementation strategy fidelity

More than half of our respondents described an opportunity for synergy between the development of implementation strategy fidelity and mechanisms-focused implementation research. While the majority of participants agreed on the importance of integrating strategy fidelity assessment within mechanisms-focused research, only two commented on how they might assess strategy fidelity, and those who did proposed differing approaches (prospectively vs. retrospectively). When asked how implementation strategy fidelity assessment fits within a mechanistic framework, one participant illustrated their thoughts with the example of a video-based health education strategy:

If the mechanism is through delivering information in an exciting and emotionally relevant way, that prompts integration of information into people…I would say that fidelity to this strategy to me would be a precondition for the mechanism activation, that’s where I would think of it…And I’m sure that there are others, well, precondition or [cognitive] moderator... probably both [cognitive] moderators and preconditions, that’s probably where I would look at some of this implementation strategy fidelity.

In this example, the participant describes a pathway where a video-based health education strategy targets the activation of new information. They went on to explain that the “people” described above referred to a group of patients in a clinic waiting room who were shown a video to improve their knowledge of a pharmaceutical drug intervention. The participant describes how adequate fidelity to the video strategy is required to activate the mechanism of new information in patients regarding the intervention. Mechanistic models categorize two constructs that can impact the relationship between a strategy and the activation of a mechanism: preconditions for mechanism activation, and cognitive moderators. Preconditions include facets of the strategy that are required for a mechanism to be activated [27]. The participant in the quote above went on to explain how clinics in their study sometimes experienced power outages, preventing patients from seeing the video. They explained how assessing the proportion of clinic days without electricity could serve as an implementation strategy fidelity indicator that might be assessed throughout the study period. Cognitive moderators are factors that impact the level of a strategy’s influence on the activation of a mechanism [27]. The participant quoted above went on to describe various cognitive moderators that might impact the video’s ability to activate the mechanism of new knowledge within a patient in the waiting area. For example, they described how a patient’s mood might impact their ability to connect with the video and process the information it was meant to deliver. They described how assessing cognitive moderators like patients’ moods while exposed to the video in the waiting room might represent important information regarding the fidelity with which the strategy was delivered. The participant also described how one might determine cognitive moderators or preconditions of mechanism activation at the outset or early stages of a study, allowing for their prospective assessment throughout the study period.

A different participant similarly described adequate implementation strategy fidelity as a requirement of mechanism activation but shared a differing view on how it might be assessed. The participant used an example where a didactic training strategy targeted the mechanism of new knowledge in a group of primary care physicians to improve their administration of a depression screening tool, with the end goal of increasing the screening tool’s uptake in their routine clinical practice. When asked how they might go about assessing implementation strategy fidelity in their example, this participant described how the activation of new knowledge and skills might be pragmatically assessed via a pre- and post-training test, a proximal indicator of that mechanism’s activation. They described how knowledge test scores might vary based on fidelity components related to the training itself (e.g., quality of delivery, coverage of content, participant responsiveness), but noted that these facets are often harder to comprehensively assess compared to something like a pre-post knowledge test. This participant suggested that if researchers find that a strategy impacts a proximal outcome, such as new knowledge and skills, they might conclude that the necessary criteria for activation were met, providing a sense that fidelity may have been adequate. To that end, the participant also described the importance of implementation strategy specification in facilitating an explanation of exactly what activities occurred leading up to the activation of a mechanism as well as clearly stating how an activated mechanism might overcome a specific implementation barrier. While nearly all researchers described the importance of integrating fidelity within mechanisms research, only the two highlighted here described how they might do so.

### Barrier 4: Pragmatic solutions to structural funding and reporting barriers

Nearly all researchers described the same structural barriers to implementation strategy fidelity assessment and reporting: word limit constraints, a lack of reporting requirements, and insufficient funding. Several researchers highlighted some journals’ more recent adoption of the Standards for Reporting Implementation Studies (StaRI) Statement as a reporting guideline [28], which they saw as a structural solution to improving implementation strategy fidelity assessment and reporting. StaRI gives researchers specific guidance and provides examples for including information about implementation strategy fidelity within implementation trials. While this seemed like a direct solution to a structural barrier, one participant voiced concern over their utility in practice:

Is [implementation strategy fidelity reporting a] common practice in the field? Heck no. I do think that, as the journals are starting to require checklists like StaRI or other things, that hopefully will become a little bit more. But I do think that journals sort of say ‘we need this’ and then sometimes I don’t even think they check.

In addition to word limit constraints and reporting requirements, several participants described the structure of funding opportunities as a barrier to implementation strategy fidelity assessment, specifically requirements related to the assessment of clinical outcomes. All researchers described costs associated with implementation strategy fidelity data collection as a barrier; several clarified further how the requirement of clinical outcome measurement drew resources that might otherwise be used to elucidate implementation strategy fidelity:

So, you know, you can’t be saying ‘I’m going to run a trial and it is going to run over three years it’s going to cost you, you know $10 million or whatever.’ Because to be looking at fidelity in a huge amount of detail? This isn’t a cost-effective study to propose. So I think, by trying to be pragmatic we lose the ability to go into a huge amount of depth on the fidelity question. So if we have more studies, with an implementation orientation…so you don’t collect any effectiveness data, that creates the space to say okay we’re going to look at scale up measures, we’re going to look at uptake, we’re going to look at the definitive feasibility, you know?

About half of all participants described working within the confines of current grant funding mechanisms, offering what they felt like were pragmatic solutions focused on reducing the costs of data collection techniques to make space within limited budgets for implementation strategy fidelity assessment. These techniques included technological innovations and finding multiple uses for data sources. Participants described the use of meta-data related to facilitator email response times and using machine learning and artificial intelligence to rate fidelity of training strategies. Several others described how costing data were regularly collected for cost-effectiveness analyses and how techniques like time and motion tracking could also be used to assess facets of fidelity to some implementation strategies (e.g., the frequency or duration of facilitator phone calls).

Despite barriers related to the operationalization of implementation strategy fidelity, the complex nature of multifaceted strategies, the assessment of implementation strategy fidelity within mechanisms research, and several challenges related to publication and funding, researchers in our sample held an overwhelming optimism and motivation towards the improvement of implementation strategy fidelity assessment and reporting. One participant described their motivation to scale up implementation strategy fidelity and reporting with a sense of pragmatism straightaway, eschewing the need to compare standards between implementation strategy fidelity and other perhaps more developed forms of measurement.

I think right now we’re at a place, we just need to start doing something. It doesn’t have to be perfectly, psychometrically, 100%, you know? We start where we are. Let’s start with the yes/no’s and the ‘did it happens?’ And then progress from there, maybe to quality and intensity and things like that… Just start where we are.

## Discussion

Participants described barriers to implementation strategy fidelity assessment and reporting in four main ways: (1) approaches to implementation strategy fidelity assessment, (2) implementation strategy complexity, (3) the role of fidelity within mechanisms-focused research, and (4) structural publication and funding barriers. Each category also included participants’ suggested pragmatic solutions. In this section, we discuss the implications of each theme (combining the first two categories due to their conceptual overlap) and contemplate a way forward.

While nearly all participants shared the same basic definition of implementation strategy fidelity, responses varied regarding its operationalization as an outcome assessed via standardized tools, versus a more descriptive assessment utilizing process data.

Several recommendations in the implementation literature have centered on the importance of specifying and tracking implementation strategies prospectively, while recording changes and deviations that adhere to reporting standards [29–33]. This type of specification and tracking documentation, as well as suggestions from participants to utilize costing or time and motion data, overlap with key components of fidelity assessment (e.g., assessing frequency, coverage, duration) [34]. Participants in our study also described the interpersonal relationships between actors and action targets as hallmarks of more complex implementation strategies. While they shared various thoughts on how the fidelity of those relationships might be assessed (e.g., adapting existing quantitative scales, qualitative interviews), their descriptions seemed to focus on assessing the quality of an actor or participant’s responsiveness—two more components of fidelity assessment [34]. In the absence of validated fidelity tools, the utility of specification and tracking data, imbued with some assessment of quality and participant responsiveness when necessary, seem consistent with fidelity theory to assess the plausibility of a Type III error in implementation research [34]. Several participants described how some interventions and implementation strategies share conceptual similarities potentially presenting opportunities to adapt intervention fidelity tools for the purpose of assessing implementation strategy fidelity. For example, fidelity to Assertive Community Treatment (ACT), a team-based intervention meant to reduce the amount of time adults with serious mental health conditions spend in hospital settings, can be assessed using the Tool for Measuring Assertive Community Treatment (TMACT) [35]. Given the team-based nature of the intervention, several TMACT items assess the presence of key staff and the amount of time spent in their roles [35]. Such items might be adapted to assess fidelity components of implementation strategies looking to create new clinical teams.

Mechanisms of implementation strategies responsible for changes in outcomes center prominently in recent implementation literature and echoed through our interviews as well [27, 36–39]. Mechanisms are defined as the processes or events through which an implementation strategy operates to affect desired implementation outcomes [40]. Participants described a synergy between implementation strategy fidelity and mechanisms development, but only two mentioned how they might go about this. While the lack of responses on this topic may reflect a current literature gap, a recent publication may serve as an example of this synergistic relationship. Larson et al. (2021) carried out a study of a motivational implementation strategy meant to activate self-efficacy and volitional mechanisms to ultimately improve the adoption, fidelity, and sustainment of an evidence-based education intervention [41]. The researchers developed an implementation strategy fidelity tool prospectively that highlighted several strategy components and assessed their fidelity using a mix of observations and recordings rated through Likert-type responses and ultimately reported adequate overall fidelity. Despite their rigorous approach, the authors note that they were unable to tease apart the impact of specific implementation strategy components on specific mechanisms due to the strategy’s blended approach. The authors call for more robust fidelity assessments alongside larger sample sizes and more complex study designs in future research to aid in such a pursuit. This work may serve as a model to further our understanding of how to best assess fidelity of implementation strategies within mechanism-focused studies. Future research may work to identify and assess fidelity components within mechanism models using prospective and/or retrospective approaches to better understand their impact on mechanism activation and proximal or distal outcomes.

The fourth theme focused on structural barriers regarding grant proposals and publication requirements. Barriers described by our participants tracked closely with our initial literature search of barriers to intervention fidelity and included manuscript and proposal word limits, the adoption and enforcement of reporting guidelines, and a strain on data collection costs related to funders’ focus on clinical outcomes. Some solutions also mirrored those proposed in the literature by champions of intervention fidelity reporting including the adoption and enforcement of reporting guidelines. Several participants additionally suggested the use of technology or the repurposing of data sources to mitigate fidelity data collection costs. Reporting guideline adoption varies across journals even though their adoption improves the quality of published research [42]. Leading implementation journals have published on the development of implementation-focused reporting guidelines like StaRI, which clearly defines and provides examples of fidelity assessment of implementation strategies [43, 44]. Similar to publishers’ adoption of intervention fidelity inclusive reporting guidelines, the adoption and enforcement of guidelines like StaRI among journals that publish implementation research may facilitate the reporting of implementation strategy fidelity.

Some participants also discussed how grant funding tends to focus on clinical outcomes, drawing resources that might otherwise be used to study implementation strategy fidelity. While the drivers of research funding priorities are complex, the impact of strategic and targeted funding leads to higher quality and quantity of publications [45–47]. As some in our sample suggested, funding targeted specifically at the development of implementation strategy fidelity assessments may accelerate the field’s understanding and achieve the end goal some stated for strategy-specific tools with strong psychometric and pragmatic properties. While working within the structure of current funding mechanisms, several participants suggested data collection techniques that might mitigate research costs and allow for more efficient fidelity data collection. Efforts to improve intervention fidelity have utilized technological innovations and other means to facilitate efficient data collection [48, 49]. It is possible that similar techniques could be applied at the level of the implementation strategy.

Our findings should be interpreted alongside several limitations including our sample’s geographic homogeneity and the virtual interview format. While our initial sample included several researchers based in sub-Saharan Africa, South America, and Europe, our final sample yielded only 1 participant based outside the USA. Not wanting to overburden researchers in our approach, we set an enrollment procedure focused on sending no more than 3 unanswered recruitment emails before discontinuing our pursuits. Although their perspectives would have been highly valued, we ultimately decided to conclude data collection after the 22 interviews described above reached a point of saturation. It is possible that the inclusion of researchers from different settings could have impacted our results and that our results may not apply as readily to research conducted outside of the USA. Due to challenges with the COVID-19 pandemic and study budget limitations, our team opted to interview participants via video conference. Qualitative researchers have described face-to-face interviews as a gold standard for data collection. Disadvantages of the virtual format include connection issues that might negatively impact audio or video quality and a reduced ability to read body language, and advantages include the ability to connect under circumstances that preclude in-person data collection [50, 51]. Despite these disadvantages, our team felt the advantages were greater given the state of the pandemic and geographic diversity of our sample within the USA.

Our study is the first to our knowledge to focus on barriers and solutions to implementation strategy fidelity assessment and reporting. We believe that our sampling procedure yielded participants who represented a high implementation research pedigree, many of whom are viewed as leaders of the field. In focusing our interview guide on pragmatic solutions in addition to barriers, our work also provides a potential way forward for the field in both the short and long term.

## Conclusion

We believe the importance of assessing and reporting implementation strategy fidelity is high given its critical role in interpreting research findings. Our respondents described a range of implementation strategy fidelity data collection and analysis techniques from their own work. At the same time, participants described how this information was often left out at the reporting stage despite its importance. In the current research landscape, publishing anything related to the assessment of implementation strategy fidelity in line with suggestions made here is likely to advance the field. To borrow from one participant, such action may serve as the most pragmatic solution to “start where we are,” with whatever we have available.

## Data Availability

Data sharing is not applicable to this article as no datasets were generated or analyzed during the current study

## Abbreviations

TDF: Theoretical domains framework
NIH RePORTER: National Institutes of Health Research Portfolio Online Reporting Tools
CIHR: Canadian Institutes of Health Research
ART: Antiretroviral therapy
StaRI: Standards for Reporting Implementation Studies
ACT: Assertive Community Treatment TMACT: Tool for Measuring Assertive Community Treatment
